# Optimized dithranol-imiquimod-based transcutaneous immunization enables tumor rejection

**DOI:** 10.3389/fimmu.2023.1238861

**Published:** 2023-09-01

**Authors:** Ann-Kathrin Hartmann, Joschka Bartneck, Jonas Pielenhofer, Sophie Luise Meiser, Danielle Arnold-Schild, Matthias Klein, Michael Stassen, Hansjörg Schild, Sabine Muth, Hans Christian Probst, Peter Langguth, Stephan Grabbe, Markus P. Radsak

**Affiliations:** ^1^ IIIrd Department of Medicine – Hematology and Oncology, University Medical Center of the Johannes Gutenberg-University, Mainz, Germany; ^2^ Biopharmaceutics and Pharmaceutical Technology, Institute of Pharmacy and Biochemistry, Johannes Gutenberg-University, Mainz, Germany; ^3^ Institute of Immunology, University Medical Center of the Johannes Gutenberg-University, Mainz, Germany; ^4^ Research Center for Immunotherapy (FZI), University Medical Center of the Johannes Gutenberg-University, Mainz, Germany; ^5^ Mainz Research School of Translational Biomedicine (TransMed), University Medical Center of the Johannes Gutenberg-University, Mainz, Germany

**Keywords:** trancutaneous immunization, imiquimod (IMQ), dithranol (anthralin), cytotoxic T lymphocytes (CTL), tumor rejection

## Abstract

**Introduction:**

Transcutaneous immunization (TCI) is a non-invasive vaccination method promoting strong cellular immune responses, crucial for the immunological rejection of cancer. Previously, we reported on the combined application of the TLR7 agonist imiquimod (IMQ) together with the anti-psoriatic drug dithranol as novel TCI platform DIVA (dithranol/IMQ based vaccination). In extension of this work, we further optimized DIVA in terms of drug dose, application pattern and established a new IMQ formulation.

**Methods:**

C57BL/6 mice were treated on the ear skin with dithranol and IMQ-containing ointments together with ovalbumin-derived peptides. T cell responses were determined by flow cytometry and IFN-ɤ ELISpot assay, local skin inflammation was characterized by ear swelling.

**Results:**

Applying the adjuvants on separate skin sites, a reduced number of specific CD8^+^ T cells with effector function was detectable, indicating that the local concurrence of adjuvants and peptide antigens is required for optimal vaccination. Likewise, changing the order of dithranol and IMQ resulted in an increased skin inflammatory reaction, but lower frequencies of antigen-specific CD8^+^ T cells indicating that dithranol is essential for superior T cell priming upon DIVA. Dispersing nanocrystalline IMQ in a spreadable formulation (IMI-Sol+) facilitated storage and application rendering comparable immune responses. DIVA applied one or two weeks after the first immunization resulted in a massive increase in antigen-specific T cells and up to a ten-fold increased memory response. Finally, in a prophylactic tumor setting, double but no single DIVA treatment enabled complete control of tumor growth, resulting in full tumor protection.

**Discussion:**

Taken together, the described optimized transcutaneous vaccination method leads to the generation of a strong cellular immune response enabling the effective control of tumor growth and has the potential for clinical development as a novel non-invasive vaccination method for peptide-based cancer vaccines in humans.

## Introduction

Vaccination is one of the most important medical interventions in the modern history, so far mainly by the prevention of formerly lethal or disabling infectious diseases. Moreover, immunotherapeutic approaches in the treatment of cancers have been tremendously successful over many tumor entities in the last decade, and here vaccination approaches are gaining more and more interest to specifically shape immune responses to further enhance therapeutic efficacy and concurrently reduce immune mediated side effects. This is achieved by the generation of high-quality tumor-specific T cells attacking and eliminating tumor cells (1). In this context, the non-invasive application of vaccines onto the intact skin also called transcutaneous immunizations (TCI) is a promising novel approach. TCI does not require injections and trained medical personal for application. In this regard, self-medication is conceivable and needle-borne accidents and therefore infections are avoided, a major concern of the WHO due to medical and socio-economic consequences (2–4). In contrast to needle-based conventional vaccines, TCI aims to target skin-resident professional antigen-presenting cells (APC) priming high quality T cell responses in draining lymph nodes.

Recently, we established a TCI method using the Toll-like receptor 7 (TLR7) agonist imiquimod (IMQ) together with the anti-psoriatic drug dithranol as adjuvants for TCI, termed DIVA (dithranol IMQ based vaccination). Unlike previous approaches based on IMQ alone (IMQ TCI) (5–8), DIVA overcomes the disadvantages of an insufficient memory response and excess skin area needed for immunization, both obstacles for the subsequent translation into the human system (9) The defined antigen specificity facilitates the targeting of APCs, and the non-invasive usage of clearly defined adjuvants makes DIVA an ideal vaccine (10). In our current work, we further refined the DIVA protocol by defining the optimal application modes as well as dosages of peptide antigen and adjuvants to induce a maximal T cell expansion and functionality. Applying the DIVA twice as a secondary boost, massively increased the frequency of antigen-specific T cells and their effector function, respectively, leading to robust T cell memory formation (DIVA^2^). In addition, we further optimized the nanocrystalline IMQ formulation to a spreadable redispersed solid nanoemulsion termed IMI-Sol+ with comparable immunological effects and advantageous storage and handling properties. This optimized DIVA was assessed in a prophylactic MC38mOVA tumor model enabling full clearance of MC38mOVA tumor cells.

In summary, the optimized needle free transcutaneous immunization method DIVA^2^ generates a robust T cell memory response capable of the effective tumor rejection and represents a novel vaccination method for peptide-based cancer vaccines suitable for further translation into the human system.

## Results

### Dithranol-IMQ-based transcutaneous immunization– optimized protocol and application

Recently, we characterized the novel immunization method DIVA based on the unique combination of the two adjuvants IMQ and dithranol, suitable for the induction of antigen-specific memory T cell responses (9). DIVA induces memory T cells solely by non-invasive application on native skin. In continuation of this work, we wanted to examine whether the application sequence of the adjuvants and peptides and their dose could be optimized further boosting T cell activation.

**Figure 1:**
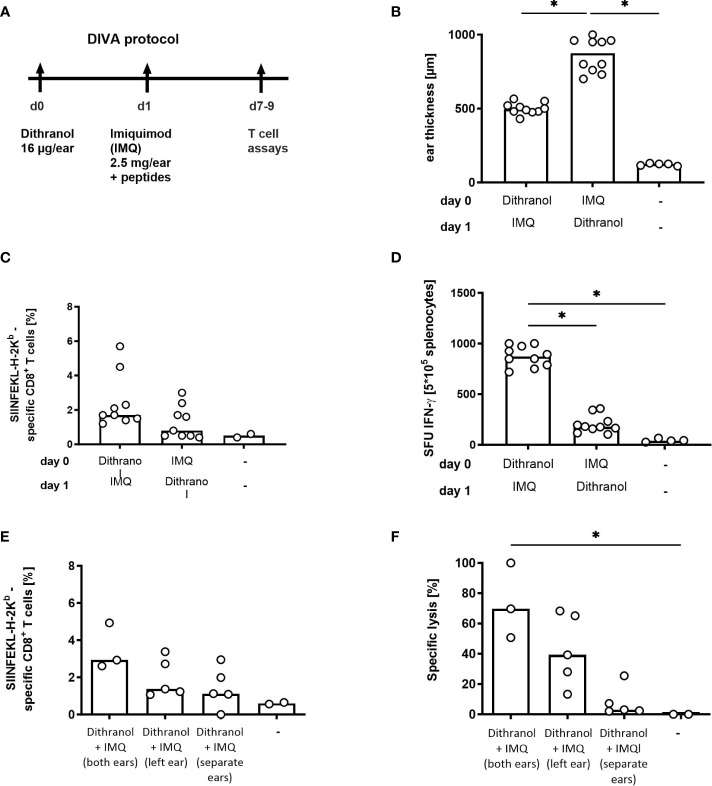
Optimization of DIVA in terms of application order in a single treatment. **(A)** Mice were immunized on both ears with dithranol petrolatum (25 mg [0.6 µg/mg) and IMQ (2.5 mg imiquimod/ear) together with SIINFEKL (OVA_257-264_) and OVA_323-337_ as indicated or left untreated. **(B)** Ear thickness was determined on day 8 using a micrometer (n= 5-10). **(C)** On day 7, the frequency of SIINFEKL-specific CD8^+^ T cells was determined in the blood of the mice using tetramer-staining (n=9, n=2 untreated) and **(D)** the effector function of the peptide-specific T cells was assessed after peptide restimulation for 20 h in an ELISpot assay (n=10, n=4 untreated). **(E)** Mice were immunized on one ear or on both ears as indicated with dithranol petrolatum (16 µg dithranol/ear) on day 0 and IMQ (2.5 mg imiquimod/ear) together with SIINFEKL (OVA_257-264_) and OVA_323-337_ on day 1 or left untreated and frequency of SIINFEKL-specific CD8^+^ T cells was assessed on day 7 in the blood of the mice (n=2-5). **(F)** Cytolytic activity of the specific T cells was determined 20h after adoptive transfer of SIINFEKL-labelled spleen cells (n=2.5). Shown are the median values (bars) with the individual measured values of the animals. *p < 0.05 by Kruskal-Wallis test and Dunn’s post-test.

Firstly, we asked whether the order of application for the adjuvants is relevant. Therefore, we treated the ears of C57BL/6 mice first with dithranol and afterwards with IMQ (as in DIVA) or vice versa and observed the induced ear swelling as well as the frequency of antigen-specific T cells in the primary immune response. Interestingly, one week after treatment ear swelling was significantly increased after the reversed application order (IMQ-dithranol) compared to the application of dithranol followed by IMQ (**Figure 1B**) indicating an augmented local inflammatory reaction. In contrast, the median frequency of antigen-specific T cells was reduced (0.8%) after IMQ-dithranol sequence and lower compared to dithranol-IMQ application (1.7%) (**Figure 1C**). While the CTL frequency displayed no statistically significant difference, IFN-ɤ production was strongly enhanced in mice immunized with dithranol-IMQ in comparison to IMQ-dithranol (**Figure 1D**), thus demonstrating that the application of dithranol prior to IMQ is important for the induction of a functionally relevant CTL response upon DIVA along with alleviating local skin irritation.

To further reduce skin irritation, it was investigated whether both adjuvants need to be colocalized at the same skin site. Therefore, we compared the application of DIVA as described before with single application of the two adjuvants on two distinct skin sites. Mice were treated with dithranol on the left ear followed by IMQ the next day on the right ear and analyzed for primary T cell responses (**Figure 1A**). As shown in **Figures 1E, F**, performing DIVA on only one ear, and thus decreasing the treated skin area by 50% resulted lower specific CTL frequencies along with a 50% reduction in specific target cell lysis *in vivo*. This suggests that the treated skin area directly correlates with the specific cytolytic immune response upon DIVA. Beyond this, when dithranol and IMQ were applied on separated skin areas, no specific CTL response was detectable (**Figures 1E, F**), indicating that both adjuvants crucially require local concurrence of both compounds for the induction of a potent immune response upon DIVA.

**Figure 2:**
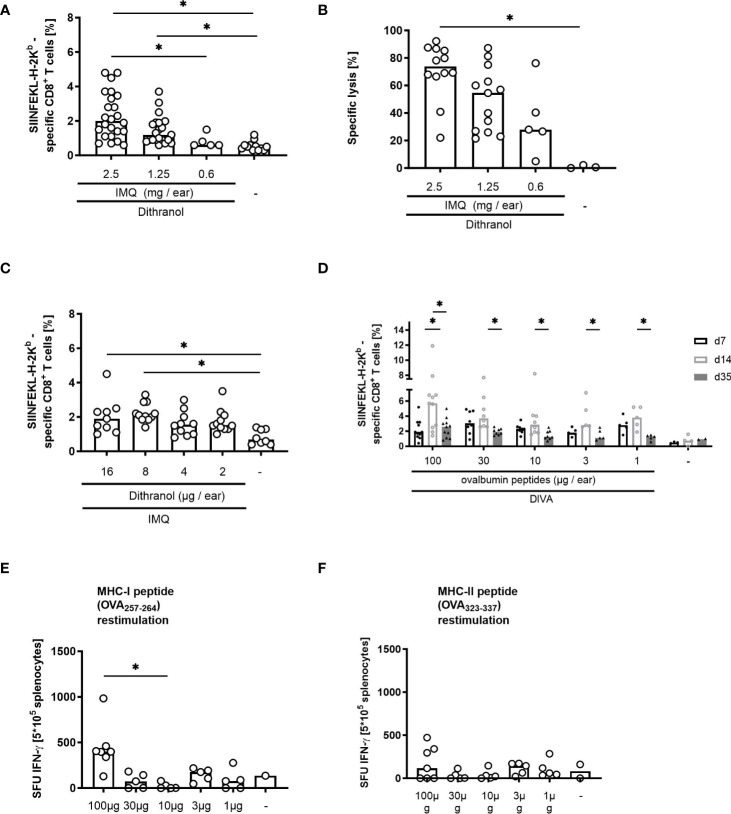
Optimization of DIVA in terms of application dosis in a single treatment. **(A)** Mice were immunized with 16 µg dithranol dispersed in petrolatum [25mg, 0.6 µg/mg w/w] and different amounts of imiquimod ointment (2.5 - 0.5 mg imiquimod/ear) together with SIINFEKL (OVA_257-264_) and OVA_323-337_ or left untreated. On day 7, the frequency of SIINFEKL-specific CD8^+^ T cells was determined in the blood of the mice using tetramer-staining (n=4-13, n=3 untreated). **(B)** the cytolytic activity was determined 20 h after adoptive transfer of peptide-labelled target cells (n=4-13, n=3 untreated) **(C)** Mice were immunized as indicated with different doses of dithranol dispersed in petrolatum [16-2 µg dithranol/ear] on day 0 and IMQ (2.5mg imiquimod/ear) together with SIINFEKL (OVA_257-264_) and OVA_323-337_ on day 1 or left untreated and frequency of SIINFEKL-specific CD8^+^ T cells was assessed on day 7 in the blood of the mice (n=2-5). **(D)** Mice were immunized with dithranol petrolatum [16 µg dithranol/ear] and imiquimod ointment (2.5 mg imiquimod/ear) together with different amounts of ovalbumin peptides (SIINFEKL (OVA_257-264_) and OVA_323-337_, 1-100 µg each) or left untreated. On day 7, 14, and 35, the frequency of SIINFEKL-specific CD8^+^ T cells was determined via tetramer staining of the blood (n=5-16) and on day 35 the effector function of the CD8 T cells was assessed in an ELISpot assay after restimulation of splenocytes with **(E)** OVA_257-264_ or **(F)** OVA_323-337_ peptide for 20 h (n=2-6). Shown are the median values (bars) with the individual measured values of the animals. *p < 0.05 by Kruskal-Walli’s test and Dunn’s post-test.

To further define the required dosages for the synergistic effect of dithranol and IMQ in DIVA, we used titrated amounts IMQ or dithranol in DIVA, respectively, and subsequently analyzed the primary immune response. As depicted in **Figure 2**, the administration of IMQ at 2.5 mg per ear was sufficient to induce a median frequency of antigen-specific T cells at about 2% with a reduction decreasing the amounts of IMQ (**Figure 2A**). This was paralleled for the specific cytolytic activity defining an amount of about 600 µg IMQ per ear as minimal dose for the induction of a detectable CTL response upon DIVA (**Figure 2B**) and demonstrates that the amount of IMQ correlates with the frequency and activity of antigen-specific T cells. For dithranol, we found that 16 µg or 8 µg per ear were necessary for the optimal induction of a primary immune response characterized by a significantly increased frequency of antigen-specific T cells (**Figure 2C**).

Finally, we also sought to define the optimal amount of antigen required for the induction of T cell responses upon DIVA and titrated the peptide amounts. As we hypothesized that the antigen dose during the priming will impact not only the primary immune response, but also memory formation (discussed in detail in (11)), we assessed the CTL frequency not only at 7- or 14-days post DIVA, but also after 35 days by *ex vivo* ELISpot assay without prior *in vitro* expansion. For the primary CTL response, we were able to detect antigen-specific T cells in the peripheral blood applying as little as 1 mg peptide onto the skin (**Figure 2D**), indicating that only minute amounts of peptide must pass the skin barrier to prime a specific CTL response at the peak to expansion 14 days post DIVA. However, to induce a persistent memory response peptide amounts of at least 30 µg were required (**Figure 2D**). Investigating functionality of the memory T cells by IFN-ɤ ELISpot assay, we used OVA_257-264_ or OVA_323-337_ peptides to detect specific CD8- or CD4-T cell activities in the memory phase. Here, the highest IFN-ɤ production by T cells was observed after immunization with 100 µg OVA_257-264_ peptide (**Figure 2E**) or 100 µg OVA_323-337_ peptide (**Figure 2F**). Different from our previous publication (9) the IFN-ɤ production is apparently different, most likely due to technical reasons.

In summary, for an optimal immune response, the efficacy of DIVA relies on dithranol and IMQ in a dose dependent manner. While both adjuvants as well as peptide antigen can be effective already at minute amounts to induce a primary specific immune reaction and concurrently reduce local skin irritation, at least 30 µg peptide antigens are required for the generation of effector memory T cells.

### IMQ formulated in IMI-Sol+ is suitable for dithranol-IMQ-based transcutaneous immunization

**Figure 3:**
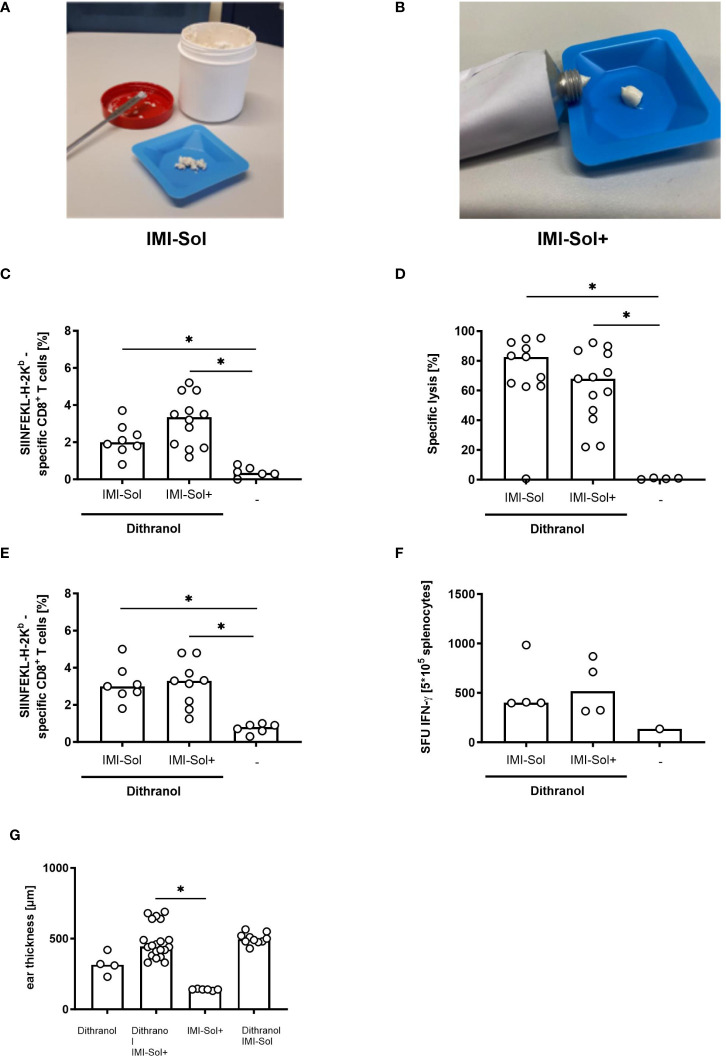
IMI-Sol+ reveals equal efficiency in DIVA approach compared to IMI-Sol. **(A)** Imiquimod nano-emulsion IMI-Sol and **(B)** IMI-Sol+. **(C)** Mice were immunized with dithranol petrolatum [16 µg dithranol/ear] and the two different imiquimod ointments IMI-Sol or IMI-Sol+ (2.5 mg imiquimod/ear) together with SIINFEKL (OVA_257-264_) and OVA_323-337_ or left untreated. The frequencies of SIINFEKL-specific T cells were determined via tetramer staining of the blood on day 7 (n=6-12) and **(D)** their cytolytic function was assessed 20 h after adoptively transfer of peptide-labelled target cells (n=8-13). **(E)** In the memory phase on day 35 the frequencies of SIINFEKL-specific CD8^+^ T cells were determined and **(F)** the effector function was revealed after restimulation of splenocytes for 20 h in an ELISpot assay to determine specific IFN-ɤ production (n=6-9). **(G)** Ear thickness was measured after treatment with dithranol and or imiquimod formulations on day 8 (n=4-20). Dithranol/IMI-Sol values from **Figure 1A**. Bars represent median value with single values depicted as dots. *p < 0.05 by Kruskal-Walli’s test and Dunn’s post-test.

IMQ formulated in IMI-Sol is a solid nano-emulsion of crystalline IMQ of crumble consistency (**Figure 3A**) that becomes viscous upon exposure to shear force to release IMQ cargo (8, 12). However, this formulation has limited shelf life as the lipophilic components tend to oxidize and degrade, becoming hygroscopic at room temperature, thus requiring below –20°C for storage (13). Due to the shear-thinning behavior, IMI-Sol cannot be filled, stored and applied in standard aluminum tubes, which are otherwise frequently used for topical products. To increase shelf life and applicability in the light of our aim to translate DIVA for application in humans, we modified IMI-Sol to a spreadable semi-solid oleogel formulation termed IMI-Sol+ (**Figure 3B**). This was achieved by adding an oleogel consisting of aerosil, isopropyl-myristate and medium chain trigylcerides and evaluated whether this alteration would affect the efficacy of DIVA. Mice were treated with DIVA using either IMI-Sol+ or IMI-Sol together with OVA_257-264_. The generation of OVA_257-264_-specific T cells was determined in the peripheral blood as before after one week and followed up until memory phase. In the primary immune response, both formulations induced potent frequencies of antigen-specific CD8^+^ T cells (**Figure 3C**) with comparable effector functions assessed by *in vivo* cytolytic activity (**Figure 3D**). In the memory phase on day 35, a significant population of OVA_257-264_-specific T cells was detectable in both groups (**Figure 3E**) with comparable IFN-ɤ production (**Figure 3F**). DIVA-induced side effects, characterized by ear swelling, were comparable between the two IMQ formulations (**Figure 3G**). Thus, the formulation IMI-Sol+ facilitates the standardized administration of IMQ with equivalent activity in DIVA compared to IMI-Sol and improved shelf life and storage properties.

### Repeated application of DIVA boosts specific primary and memory T cell formation

DIVA induces a potent primary T cell response in a frequency of up to 10% of circulating CD8 T cells after 14 days protective against a lethal vaccinia virus challenge (9). To assess whether DIVA-induced T cell responses are limited to single application, we investigated whether it is possible to further boost the generated cellular immune response by a repeated immunization. Since we defined a minimal dose of dithranol required for the induction of a primary immune response (**Figure 2C**), we reasoned that for repeated application lower doses of dithranol or prolonged intervals between treatments might be helpful to enhance vaccination efficacy and ameliorate local skin reactions. Therefore, modulations of DIVA application scheme included the dose of dithranol (ranging from 2 to 8 µg dithranol/ear) and up to three treatment cycles as detailed in **Supplementary Figure 1A**. While in case of repeated application of DIVA more than 8 µg dithranol caused severe skin irritations (not shown), we observed a massive increase in the frequency of specific CD8^+^ T cells in the peripheral blood upon repeated application on day 14 compared to single treatment also using lower doses of dithranol (**Supplementary Figure 1B**). Interestingly, monitoring the CTL frequency over time, we found the most pronounced CTL expansion up to 60% using DIVA twice with 8 µg Dithranol (DIVA^2^) (**Supplementary Figures 1C, D**). Notably, lower doses of dithranol or an additional treatment cycle yielded lower CTL frequencies. Moreover, this strong CTL response was accompanied with transiently aggravated local skin reactions (**Supplementary Figure 1E**) indicating that repeated DIVA treatment is limited by adverse local skin reaction as well as in terms of immune cell stimulation favoring one additional vaccination for conversion to persistent memory T-cell responses.

**Figure 4:**
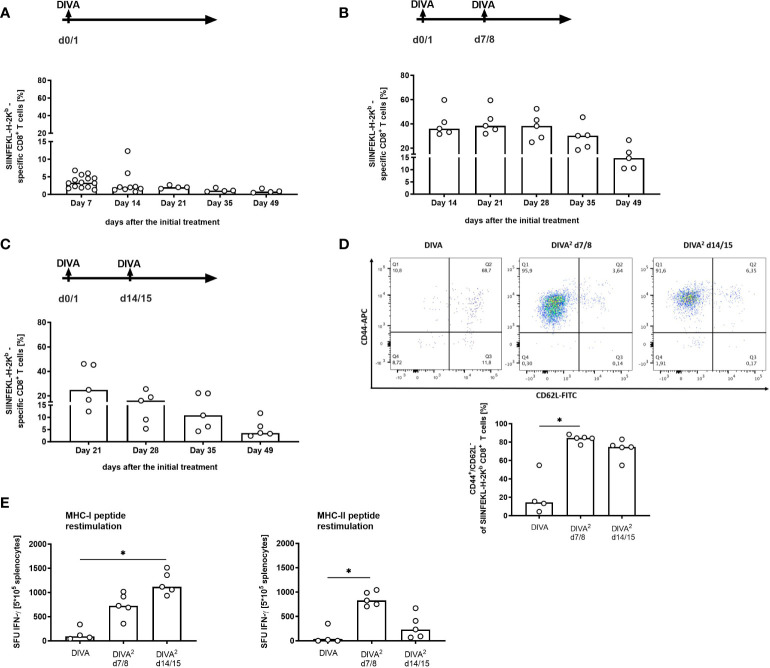
Second immunization one week after the initial treatment induces a ten-fold increased memory response. Mice were immunized with dithranol petrolatum [8 µg dithranol/ear] (day 0) and IMI-cream (2.5 mg Imiquimod/ear) together with SIINFEKL (OVA_257-264_) and OVA_323-337_ (100 µg per ear) (day 1) **(A)** in one single application (DIVA) or **(B)** boosted on day 7/8 or **(C)** on day 14/15. One week after the first treatment, tetramer staining of blood cells was performed to assess the frequency of SIINFEKL-specific CD8^+^ T cells once a week until day 49 (n=5 for each group). Bars represent median value with single values depicted as dots. **(D)** Analysis of the effector function of specific CD8^+^ T cells in the memory phase (CD8^+^, H2-Kb-SIINFEKL^+^, CD44^high^/CD62L^low^ cells). Gating strategy is depicted in **Supplemental Figure 2A**. **(E)** Restimulation of splenocytes with SIINFEKL (OVA_257-264_) and OVA_323-337_ (1µM each) for 20 h to determine specific IFN-ɤ production of memory T cells (n=2-5). Bars represent median value with single values depicted as dots. Statistical analysis by Kruskal-Walli’s test and Dunn’s post-test (*p < 0.05).

Next, we asked for the optimal interval between treatments for DIVA. Therefore, we compared DIVA as single treatment with an additional treatment cycle after 7 or 14 days. As shown in **Figure 4**, we observed that an additional treatment cycle resulted in a major increase in specific CTL frequency compared to single application. Although, single application of DIVA resulted in a considerable primary and memory CTL response, a second treatment cycle massively boosted the antigen-specific CTL response at both time points (at 7 or 14 days post priming). However, the peak expansion up to 40% peptide specific CTLs occurred upon secondary treatment after 7 days (**Figure 4B**) compared to only 20% specific CTLs after 14 days (**Figure 4C**). Notably, also IFN-ɤ production *ex vivo* and effector phenotype persisted until day 49 indicative of a durable effector memory CTL response. We also revealed the impact of the application area needed for immunization. In this context we reduced the immunization area for DIVA^2^ by treating only one ear which corresponds to an area of about 1.5 cm^2^ with different antigen doses. In this context, antigen-specific CTL response is not significantly affected by reducing the skin area by half. Additionally, we analyzed the boosting effect of DIVA^2^ when the second treatment was performed on a different skin area. In detail, mice were treated on one ear in the first immunization and boosted after 7 days on the other ear. In this context, antigen-specific CTL frequencies were not affected after high dose immunization or addition of MHC class II restricted OVA_323-337_ peptides compared to boosting the same skin side twice (**Supplementary Figure 3**). Overall, this allows the conclusion that DIVA^2^ with a secondary boost after 7 days results in superior CTL expansion and memory formation.

### Optimized DIVA^2^ mediates full protection against a lethal tumor challenge

**Figure 5:**
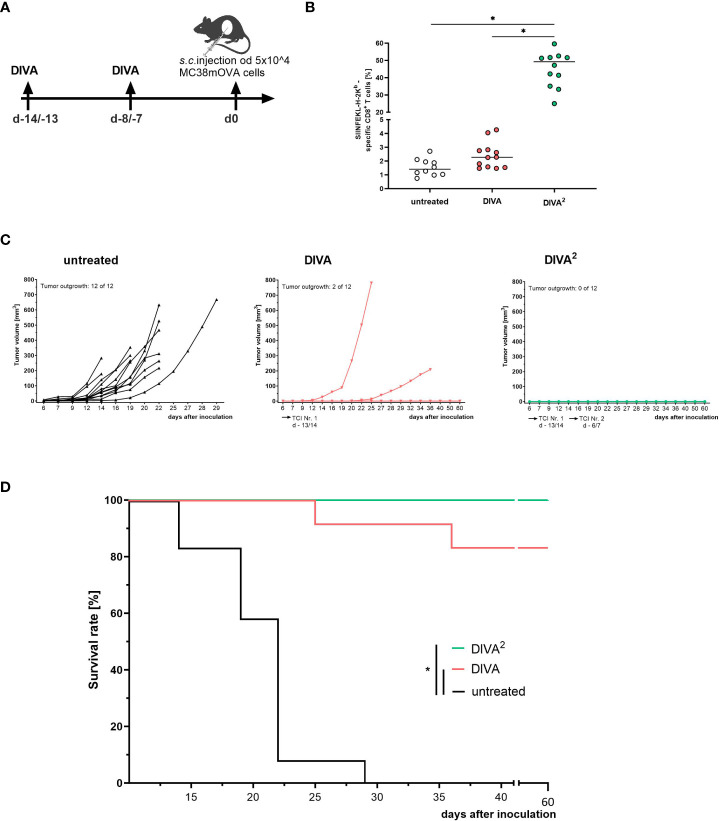
Twofold immunization completely controls tumor growth in a prophylactic tumor model. **(A)** Schematic overview of the application pattern for DIVA^2^ in a prophylactic tumor setting. On day 0 5x10^4^ MC38mOVA tumor cells were inoculated subcutaneously. **(B)** Tetramer staining of pre-immunized mice at the day of tumor cell inoculation (day 0, n=12). **(C)** Tumor volumes were assessed three times per week. Every curve represents the tumor volume of one individual mouse (n=12). **(D)** Kaplan-Meier survival curve. Statistical analysis of tetramer staining using Kruskal-Wallis with Dunn`s post-test. Comparisons of survival curves were performed by Log-rank (Mantel-Cox test) (*p < 0.05).

After establishing the optimized vaccination scheme DIVA^2^ we evaluated the biological efficacy of the induced specific T cell responses in a tumor model using MC38 colon carcinoma cells transfected to express OVA as a target neoantigen (MC38mOVA). C57BL/6 mice were immunized once or twice and subsequently challenged by subcutaneous injection of 5x10^4^ MC38mOVA tumor cells (**Figure 5A**). The vaccination efficiency of DIVA was assessed on day 0 by the frequencies of antigen-specific CD8^+^ T cells in the peripheral blood. As demonstrated before, repeated vaccination by DIVA^2^ yielded a high frequency of antigen-specific T cells and an approximately 30-fold increase compared to single administration of DIVA (**Figure 5B**). Although, single DIVA induced specific CTL frequencies up to 5%, we observed tumor outgrowth in 17% tumor challenged mice (2 of 12 mice injected with MC38mOVA). In contrast, in DIVA^2^-treated mice no tumors were detectable, thus 100% tumor protection was achieved (**Figures 5C, D**), highlighting the relevance of sufficient numbers of activated CTLs for full tumor clearance.

Taken together, these data indicate that DIVA^2^ boosts specific CD8^+^ T cell generation that converts to effective primary and memory responses that are highly functional in clearing MC38mOVA tumor cells *in vivo* in a prophylactic tumor vaccination setting.

## Discussion

Adding dithranol in DIVA to our formerly developed IMQ-based non-invasive TCI (IMQ-TCI) (8) already generates a major advantage for transcutaneous vaccination approaches, as this enables the formation of memory T-cell responses along with a significantly reduced application area (9). While dithranol and IMQ are already established agents for topical use on the skin in clinical use, the concomitant topical use of both agents has been uncommon and therefore requires careful dose titrations to balance vaccination efficacy versus adverse skin reactions that cumulate by combined use. While aiming for clinical translation to humans in perspective, evaluation of efficacy and tolerability in rodent models represents the first step for this. Therefore, we refined our formulations and immunization protocol to pave the way to an all-in-one transcutaneous immunization approach.

In this optimizing process, we confirm the order of adjuvant application in DIVA: first dithranol followed by IMQ. The best immunization efficiency along with the least side effects is achieved by applying dithranol prior to IMQ (**Figures 1B–D**). This is in line with our recent findings on the mechanisms induced by dithranol in the skin. After application of dithranol, monocytes migrate into the skin promoting inflammation crucial for formation of antigen-specific T cells (9). Furthermore, dithranol and IMQ must be applied onto the same skin site (**Figure 1F**) indicating an essential conditioning function of dithranol by attracting monocytes to the skin facilitating APC activation and T cell priming. Reducing the treated skin area results in a diminished immune response (**Figure 1F**), indicating the need of a minimal area treated for the immunization, in mice apparently both ears in a single DIVA treatment. Performing boost immunization, reducing the treated skin area by half also leads to robust memory CTL frequencies in the blood highlighting the superior effect of DIVA^2^. Boosting on two different ears also induced antigen-specific CTLs with comparable frequencies when high antigen-dose immunization was performed (100 µg) suggesting a systemic boosting effect of DIVA^2^ (**Supplementary Figure 3B**). Addressing the adjuvant doses, 2.5mg IMQ (**Figures 2A, B**) and 16 µg or 8 µg dithranol dispersed in petrolatum (**Figure 2C**) per ear induces the strongest primary immune response, corresponding to a dithranol concentration of 0.6 µg/mg or 0.3 µg/mg. This dithranol dose is within the range that Benezeder and colleagues used to assess the effect of dithranol in an IMQ-induced psoriasis model on the back skin of mice. They used 40 mg of dithranol petrolatum in increasing concentrations daily from 4-40 µg per application (0.1 µg/mg - 1 µg/mg. In contrast to our setting using single application, they also performed a mouse tail test by applying 400 µg dithranol daily (10 µg/mg) for 14 days (14). For the treatment of psoriasis, dithranol is used as a short contact therapeutic agent, initially in a concentration of 1 µg/mg -2.5 µg/mg (15). Thus, the necessary amount of dithranol in DIVA is far below the amount commonly used in animal models and in the treatment of psoriatic patients.

Booster vaccines enhance the natural immune response after the initial immunization by inducing stronger, long-lasting and more specific immune responses (16). Since dithranol leads to the formation of skin inflammation accompanied by edema (17) and hyperkeratosis (9), it is necessary to keep the dithranol concentration as low as possible in a multiple application to prevent exaggerated inflammatory reaction of the treated skin. In a single application approach, 8 µg of dithranol per ear is sufficient to induce a primary immune response (**Figure 2C**). Hence, performing boosting immunizations with decreasing dithranol doses (from 8 µg to 2 µg) and increasing immunization frequencies (**Supplementary Figure 1**) reveals a massive increase of circulating antigen-specific CD8^+^ T cells one week after the second immunization at about 60% resulting in a robust memory response which significantly differs from the untreated or the IMQ-treated mice. Here, a median frequency of about 20% antigen-specific CD8^+^ T cells was detectable in the memory phase (**Supplementary Figure 1C**; **Figure 4B**), a ten-fold increase compared to a single dose treatment (**Figures 3E** or **4A**). Furthermore, the induced memory CD8^+^ T cells show the highest IFN-ɤ-production after peptide restimulation (**Supplementary Figure 1D**), comparable to that measured during the primary immune response (**Figure 3F**). As expected, ear thickness increased concentration-dependently after a second or third application of dithranol petrolatum. Maximum ear swelling was observed after a double application of 8 µg dithranol per ear three days after the 2^nd^ treatment, however, this normalized to the original starting level in the memory phase (**Supplementary Figure 1E**). This observation is consistent with the dithranol-mediated effect on psoriatic skin. Topical application of dithranol on psoriatic-like skin in c-Jun/JunB knockout mice significantly improves the skin lesions (14). On one hand dithranol induces an inflammatory milieu in the skin by effecting keratinocyte proliferation and cytokine secretion (18, 19), on the other hand, dithranol also downregulates pre-existing inflammation, probably by influencing keratinocyte-neutrophil crosstalk (14). In a boost vaccination, the second dithranol treatment might therefore further strengthen the already existing pro-inflammatory milieu, promoting the influx of immune cells into the skin which potentiates the amount auf antigen-presenting cells (APC).

To further develop an all-in-one formulation, we established the new nanocrystalline IMQ formulation IMI-Sol+ with comparable potent immunological effects (**Figure 3**) to IMI-Sol while simplifying application and storage. IMI-Sol+ is a further development of IMI-Sol, in which the freeze-dried nano-emulsion is dispersed with isopropyl-myristate (IPM) and the gel-forming agent aerosil in a medium chain trigylcerides (MCT)- base. IPM also serves as chemical penetration enhancer, which is described to promote drug delivery (20, 21) and protein delivery (22) over the intact skin. At the same time, IPM has a nourishing property, ensures cohesion, and is widely used in cosmetics (23). The mechanisms, how IPM promotes penetration are not fully understood, but it has been reported that IPM influences the microstructure of the *stratum corneum*, the outermost layer of the skin by interacting with the lipid structures through its hydrophobic properties (24). The newly established IMQ formulation IMI-Sol+ was used in further experiments to highlight the optimal timepoint for the 2^nd^ immunization. When boost vaccination was performed one week (**Figures 4B, D**) or two weeks (**Figure 4C**) after the initial immunization, a high increase in the frequency of antigen-specific T cells was observed. This increase lasts until the memory phase. Comparing the two boost vaccination protocols, the 2^nd^ immunization on days 7 and 8 induces the strongest memory responses, reflected by a robust frequency of antigen-specific CD8^+^ memory T cells at about 20% (**Figure 4B**) and high amounts of spot-forming units in IFN-ɤ ELISpot assays after restimulation with MHC I (OVA_257-264_) and MHC II (OVA_323-337_) ovalbumin-derived peptides, reflecting the existence of high effective specific CD4^+^ and CD8^+^ memory T cells (**Figure 4E**). However, these results need to be interpreted with caution as we did not normalize the amount of MHC class II positive antigen presenting in this assay. Therefore, there might be a bias in estimating the magnitude and kinetics of CD4 T cell responses. In contrast to single DIVA treatment, CD4 T cells play a minor role in the induction of the superior CD8 memory T cell response after DIVA^2^ treatment. Immunizations with solely the MHC I epitope (OVA_257-264_) resulted in comparable frequencies of specific CD8 T cells (**Supplementary Figure 3A**). Characterization of the memory cells identifies the resulting CD8^+^ antigen-specific T cells as mostly CD44^high^/CD62L^low^ T_EM_ cells (**Figure 4D**). This observation is in line with the common model of memory T cell formation. In this model, longer or repeated antigenic stimulation favors the generation of CD62L^low^ effector T cells (11). Moreover, additional work needs to be done to further characterize the induced memory T cells. Boosting on day 14/15 results in a decrease in the frequency of antigen-specific T cells one week after the 2^nd^ immunization. This could be related to the reduced cell number after a single DIVA on day 14 (**Figure 4A**).

To assess the biological relevance of the optimized DIVA^2^ protocol, a prophylactic tumor experiment was performed mediating complete tumor rejection in a prophylactic tumor setting (**Figure 5C**) accompanied by a robust CD8 and CD4 memory response (**Figure 4E**). As CD8^+^ CTL have a major role in immunological tumor rejection, it is a major aim to boost CTL anti-tumor capacity, i. e. by checkpoint blocking agents (discussed in (25)). However, recent studies also highlight a prominent role of CD4^+^ T cells in the setting of tumor control (discussed in (26)). As most tumor cells lack expression of MHC class II, in most cases, the effect of CD4^+^ T cells is limited to their release of cytokines. However, cytotoxic CD4^+^ T cells have been also described (27). CD4^+^ T cells promote CTL function directly by secretion of IL-2 (28–30) and IL-21 (31) and indirectly by modifying the antigen presentation of APCs, resulting in higher CTL frequencies (29, 32, 33). Massive IFN−ɤ after restimulation with a CD4 epitope indicates a T_h_1 memory, a key player in the activation and maintenance of an anti-tumor immunity (34, 35). By secretion of IFN-ɤ, migration of CTLs into the tumor microenvironment (TME) is promoted (35). Therefore, CD4^+^ T-cells inhibit tumor proliferation and tumor angiogenesis (36) and also support anti-tumor function of myeloid cells (37). Currently, tumor antigen-specific CD4^+^ T cells are thought to be essential for the clinical benefit of cancer vaccines as the induced CD8^+^ CTLs alone are not sufficient (35, 38). The high frequency of antigen-specific CD4^+^ T cells after DIVA^2^ reveals DIVA^2^ as a promising tumor vaccination tool. In this regard, further work must be done to elucidate the therapeutic effects of DIVA.

For the translation into the clinical application, further development is needed to create an all- in –one transcutaneous immunization approach. In the present work, we completed the first step by generating a spreadable IMQ formulation without negatively influencing the immunological properties of the freeze-dried nano-emulsion IMI-Sol. In the next step, the required peptides and the second adjuvant dithranol should be integrated into the formulation. Due to its chemical structure, dithranol is highly sensitive to oxidation (39), representing a challenge for further formulation development. Dispersed in petrolatum, dithranol shows a satisfying stability and it has been used successfully on patients since the early 20^th^ century (40–42).

Taken together, our present work defines the optimal application scheme and dose of antigen and adjuvants for the non-invasive vaccination platform DIVA leading to tumor protective CD8 and CD4 T-cell responses in a rodent model. IMQ and its chemical variants represent promising adjuvants that are used for immunization approaches (43–46). However, to our knowledge, there is no comparable non-invasive immunization technique using IMQ and dithranol inducing comparable T cell responses in an ovalbumin vaccination model. Pharmaceutical challenges need to be taken to develop an all-in-one formulation with comparable efficacy and tolerability before translating this promising novel vaccination technique into human application to combat cancer and emerging infectious diseases.

## Materials and methods

### Mice

C57BL/6 mice - purchased from the Envigo Laboratory (Envigo, Indianapolis, USA) or were obtained from the local animal facility of the Johannes Gutenberg-University of Mainz (TARC) - were used at the age of 8-10 weeks. All animal studies were conducted according to the national guidelines and were reviewed and confirmed by an institutional review board/ethics committee headed by the local animal welfare officer of the University Medical Center (Mainz, Germany). The responsible national authority (National Investigation Office Rhineland-Palatine, Koblenz, Germany) gave approval of the animal experiments (Approval ID: AZ 23 177-07/G18-1-096).

### IMI –Sol+ manufacturing

The IMQ-containing formulation IMI-Sol was formulated as previously described (8, 12). IMI-Sol+ is produced by dispersing IMI-Sol into an oleogel formulation consisting of 7% aerosol and 10% isopropyl-myristate (IPM) in medium chain triglyceride-base (MCT). Unification of the two formulations was performed in a melanin bowl in a ratio of 1:1 and filled into aponorm aluminum tubes. The formulation can be stored at 4°C.

### Transcutaneous Immunizations with DIVA

Immunizations were performed under isoflurane/oxygen anesthesia. For single DIVA (9), mice were treated on both ears with 25 mg dithranol-petrolatum (0.6 µg/mg - 0.08 µg/mg), corresponding to a total amount of dithranol per ear of 16 µg - 2 µg. Dithranol formulations were manufactured by the Pharmacy of the UMC Mainz according to European Pharmacopeia (Ph. Eur.) standards. On the following day, mice were treated with 50 mg-125 mg of IMI-Sol formulation or IMI-Sol+ containing 5% IMQ manufactured by the group of Prof. Peter Langguth, JGU Mainz, Germany) (corresponding to a total amount of 2.5 mg - 0.6 mg IMQ per ear). Ovalbumin peptides OVA_257-264_ (SIINFEKL) and OVA_323-337_ (OTII) (concentrations as indicated in the range of 1-100 µg each, from peptides & elephants, Henningsdorf, Germany) were solved in DMSO and applied stirred in hydrophobic DAC basic cream immediately after IMQ treatment. For boost immunization, treatment was repeated after 7 or 14 days.

### Tumor cell inoculation

Prior to tumor cell inoculation, mice were immunized two times with DIVA as indicated above. For the inoculation of MC38mOVA tumor cells mice were anesthetized. 5x10^4^ tumor cells diluted in 100 μl PBS per mouse were implanted subcutaneously (*s.c.*) on the shaved right flank. After 6 days tumors were palpable. Tumor size was measured three times per week with a digital caliper and the tumor volume was calculated. The survival of the mice was monitored. The Tumor experiment was stopped when the tumor volume of a mouse exceeded 600 mm^3^ or when ulceration of a tumor was observed.

### Generation of spleen cell suspensions

At the end of an immunization experiment, mice were sacrificed, spleens were removed and homogenized over a 70 µm cell strainer with a syringe plunger. Erythrocytes were lysed in a hypotonic lysis buffer followed by resuspension in PBS. For ELISpot analysis, cells were counted using animal blood counter Scil Vet ABC™ (Scil animal care company GmbH, Viernheim, Germany) and resuspended in Iscove`s modified Dulbecco’s medium (IMDM, Gibco, Carlsbad, USA) supplemented with 10% FCS, 1% glutamine an 1% sodium pyruvate at a concentration of 2x10^7^ cells/ml). For flow cytometric analysis of the in vivo cytotoxicity, cells were resuspended in PBS supplemented with 0.5% BSA and 5mM EDTA (FACS buffer).

### Flow cytometric analysis to assess specific T cell responses

For flow cytometric analysis of DIVA-induced specific T cell responses blood samples were obtained through a puncture of the tail. Erythrocytes were lysed via hypotonic lysis with ACK buffer for 10 minutes at RT. Cells were resuspended in FACS buffer. Samples were incubated for 30 min at 4°C with fluorescently labeled antibodies against CD8 (Pacific Blue-conjugated, clone 53-6.7, BioLegend, Koblenz, Germany), CD44 (APC-conjugated, clone IM7, BioLegend, San Diego, USA) and CD62L (FITC-conjugated, clone MEL-14, BioLegend, San Diego, USA). T cells specific for -H2K^b^-OVA_-257-264_ were detected by H2-K^b^ tetramer (PE-conjugated, in-house production, Institute for Immunology, Johannes Gutenberg University Medical Center, Mainz, Germany). All analyses were performed with a LSRII Flow Cytometer and FACSDiva software (BD Pharmingen, Hamburg, Germany).

### *In vivo* cytotoxicity assay

The effector function of the antigen-specific CD8^+^ T cells was assessed by transfer of 2 x 10^7^ target splenocytes labelled with either 4 mM (CFSEhigh) or 0.4 mM (CFSElow) carboxyfluorescein diacetate succinimidyl ester. The CFSElow cells were additionally loaded with SIINFEKL-peptide (OVA_257-264_) (2 mM) for 1h at 37°C. Both populations were transferred *i. v.* in a 1:1 ratio. 20 h after *in vivo* cell transfer, blood samples or spleen cell suspensions of immunized and naïve control mice were obtained and the specific lysis of the peptide-pulsed target cells was determined by flow cytometry. All analyses were performed with a LSRII Flow Cytometer and FACSDiva software (BD Pharmingen, Hamburg, Germany).

### IFN-ɤ ELISpot assay

Peptide-specific release of IFN-ɤ was assessed as previously described (9). Briefly, 96 Well MultiScreenHTS IP plates (0.45 mm, Merck Millipore, Darmstadt, Germany) were coated over night at 4°C with murine anti-IFN-ɤ antibody (clone AN18, Mabtech, Nacka Strand, Sweden (10 μg/ml)). After a washing step with PBS, the membrane was blocked with IMDM (Gibco, Carlsbad, USA) supplemented with 10% FCS, 1% glutamine and 1% sodium pyruvate for at least 60 min at 37°C. Spleen single cell suspensions were prepared as described before. 5x10^5^ splenocytes were incubated in the absence or presence of OVA_257-264_ or OVA_323-337_ peptides (each 1 μM). After 20 h of incubation at 37° C and 5% CO_2_ the plate was washed six times with PBS including 0.05% Tween and the produced IFN-ɤ was detected with a biotinylated anti-IFN-ɤ antibody (clone R4-6A2, Mabtech, Nacka Strand, Sweden (2μg/ml)). After an additional washing step as described above, the enzyme-substrate reaction was performed to visualize the occurred binding using the Vectastain ABC Kit (Vector Laboratories, Burlingame, USA) together with AEC (Sigma-Aldrich, Taufkirchen, Germany) as described by the manufacturer. The reaction was stopped with H_2_O. After drying, spot forming units (SFU) were measured with the automated image analysis system AID iSpot ELISpot reader (AID Autoimmun Diagnostika, Straßberg, Germany).

### Statistical analysis

The statistical analysis was performed using GraphPad Prism (version 9.4.1 for Mac OS, GraphPad Software, San Diego California, USA). Multiple comparisons between more than two groups were performed with Kruskal-Wallis test and Dunn’s post-test or with two-way ANOVA and Bonferroni’s post-test as appropriate. Comparisons of survival curves were performed by Log-rank (Mantel-Cox) test. The significance level was determined as a p value </= 0.05.

### Supplemental materials

Gating strategies for flow cytometry **Figure S2**.

## Data availability statement

The original contributions presented in the study are included in the article/**Supplementary Material**. Further inquiries can be directed to the corresponding author.

## Ethics statement

The animal study was approved by National Investigation Office Rhineland-Palatine, Koblenz, Germany. The study was conducted in accordance with the local legislation and institutional requirements.

## Author contributions

Conceptualization: MR, HS, SG, PL, and MS. Methodology: A-KH, JB, DA-S, JP, SLM, and SM. Investigation: A-KH and JB. Visualization: A-KH and JB. Funding acquisition: MR, SG, and PL. Project administration: PL, SG, and MR. Supervision: HS, PL, HCP, and MR. Writing – original draft: A-KH. Methodology review: JB and MK. Writing – review & editing: MR, A-KH, and JB. All authors contributed to the article and approved the submitted version.
