# Supplementary material for: Optimized dithranol-imiquimod-based transcutaneous immunization enables tumor rejection
Source: Front Immunol. 2023 Sep 1;14:1238861. doi: 10.3389/fimmu.2023.1238861 (PMC10505723; doi:10.3389/fimmu.2023.1238861)
Supplement: Supplementary file 1 [file DataSheet_1.pdf]

frontiers

Supplementary Material

Optimized dithranol-imiquimod-based transcutaneous immunization enables tumor rejection

Ann-Kathrin Hartmann[1], Joschka Bartneck[1], Jonas Pielenhofer[2], Sophie Luise Meiser[2], Danielle Arnold-Schild[3], Matthias Klein [3‡], Michael Stassen [3‡], Hansjörg Schild[3†‡], Sabine Muth[3‡], Hans-Christian Probst[3‡], Peter Langguth[2‡], Stephan Grabbe[4†‡], Markus P. Radsak[1,*†‡]

*** Correspondence:** Corresponding Author: radsak@uni-mainz.de

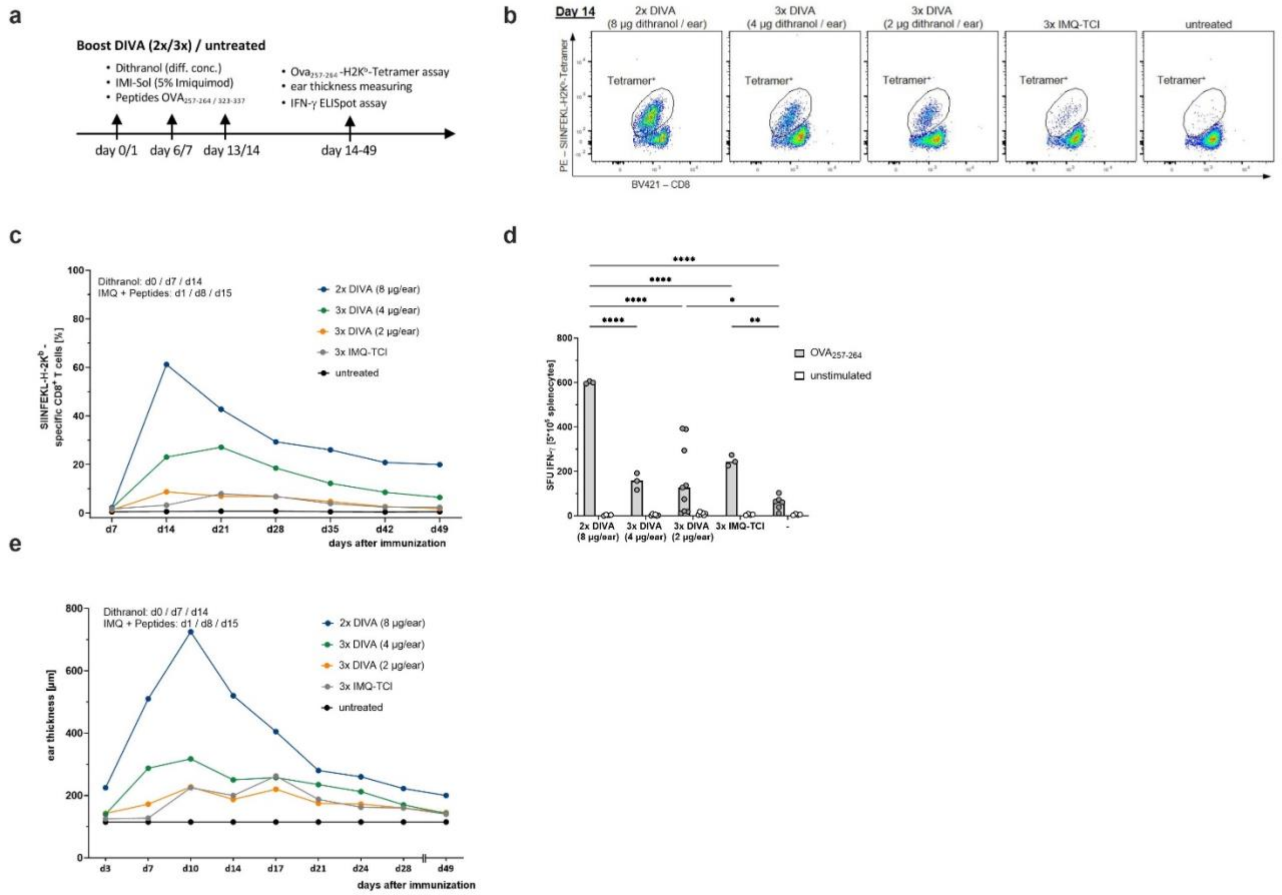

Supplementary Figure 1: Multiple DIVA application leads to a massively increased T cell response. a Schematic overview of the application pattern for multiple DIVA treatments. Mice were immunized on both ears with dithranol in petrolatum (dithranol doses as indicated [µg/ear], day 0) and IMQ (5% imiquimod [w/w]) together with $OVA_{257-264}$ and $OVA_{323-337}$ (day 1), treated only with IMQ together with peptides or left untreated. **b** Representative flow cytometry dot plots of $CD8^+$ tetramer-positive T cells after different DIVA protocols. **c** After seven days, tetramer staining was

performed to assess the frequency of SIINFEKL-specific $CD8^+$ T cells once a week until memory phase (day 49) (n=5, median). **d** Splenocytes were restimulated at day 49 with antigenic peptides for 20h in an ELISpot assay to assess specific IFN-γ production. Bars represent median value with single values depicted as dots (n=3-9). **e** Ear thickness was determined twice per week until day 28 and additionally on day 49 (n=10 ears of 5 mice, median). *$p < 0.05$ Kruskal-Wallis test and Dunn's post-test.

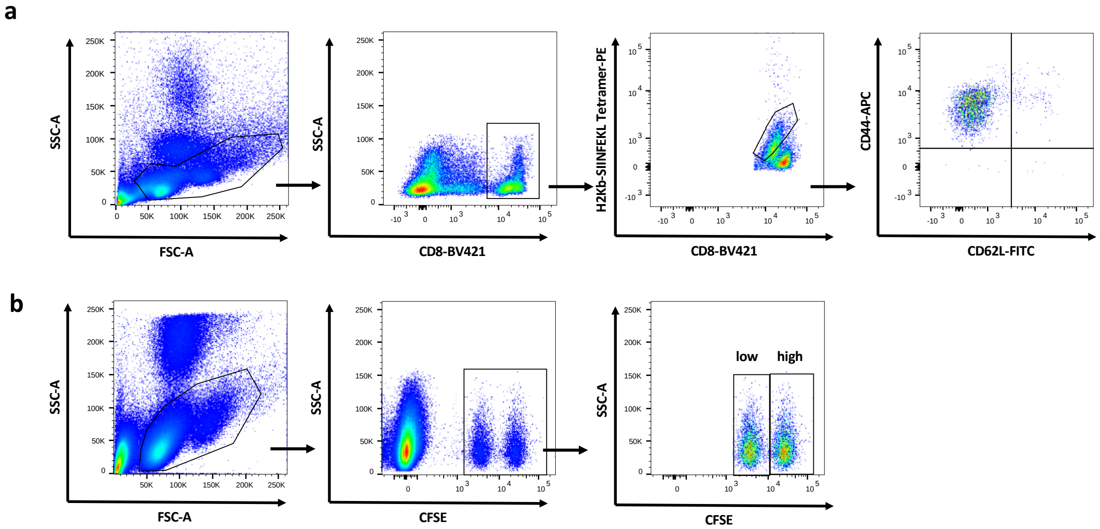

Supplementary Figure 2: Gating strategies for flow cytometry. a Gating strategy for the quantification of SIINFEKL ($OVA_{257\text{-}264}$)-specific $CD8^+$ T cells and the characterization of the activation phenotype by the expression of the activation markers CD44 and CD62L. $CD44^{high}$, $CD62L^{low}$ cells represent effector memory T cells (T_{EM}). **b** Gating strategy for the determination of the specific lysis by CTLs. Illustration of CFSE-labelled cells in spleen samples of control mice.

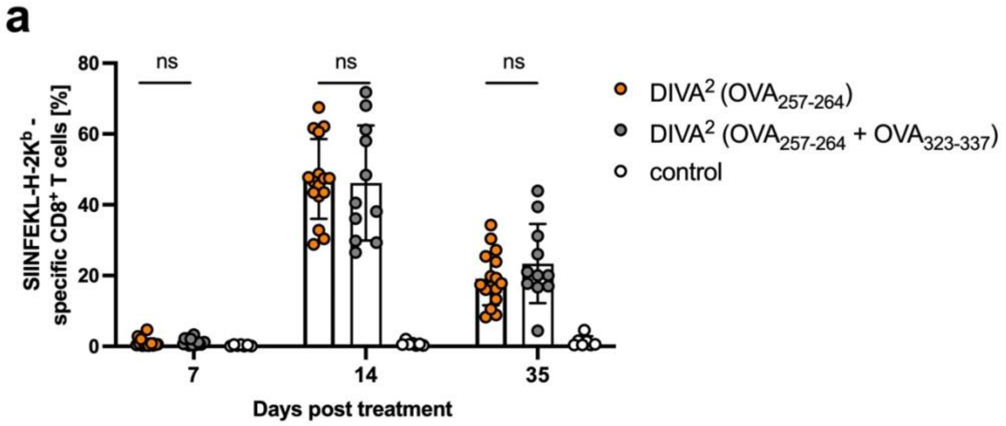

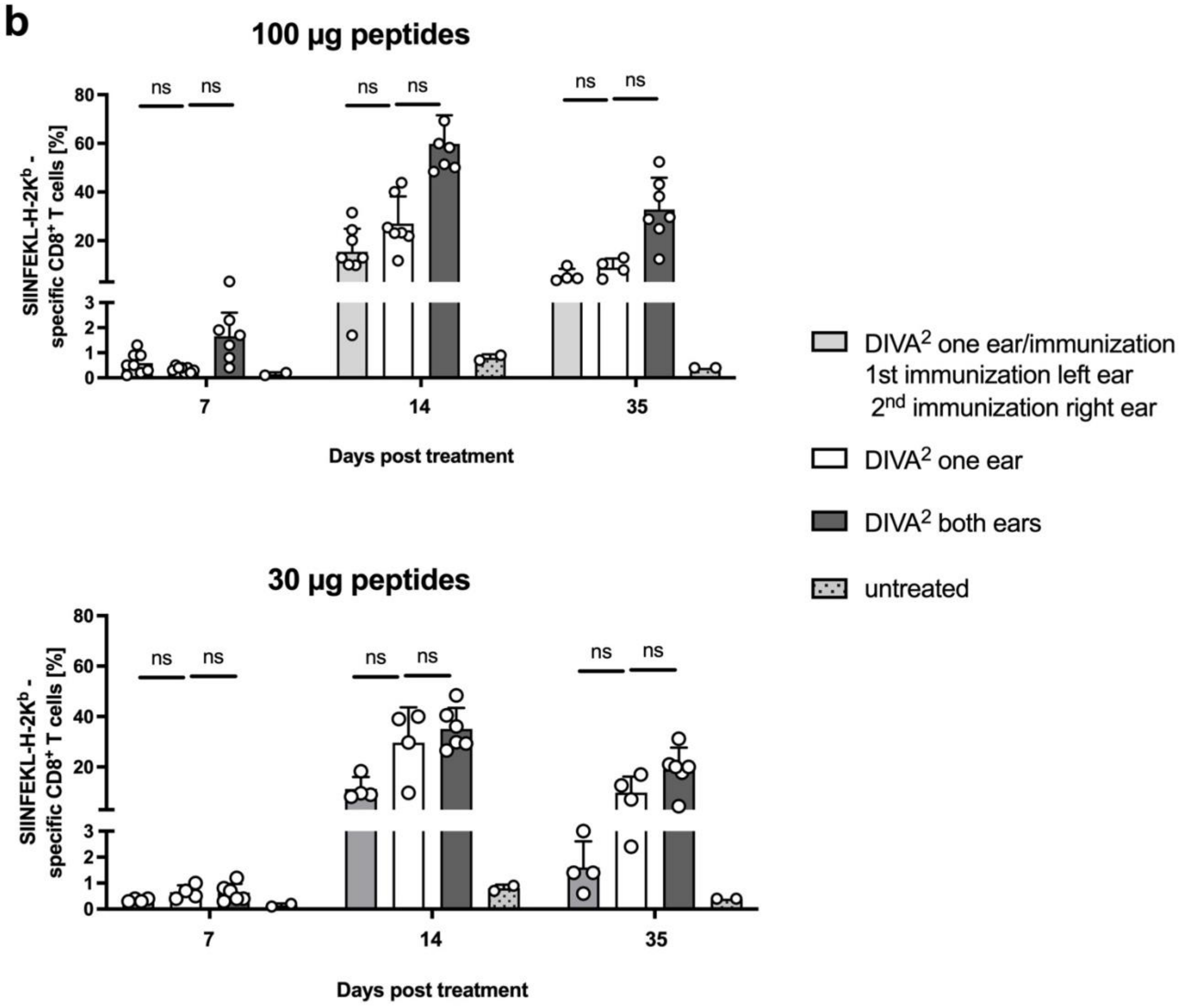

Supplementary Figure 3: Analysis of the induced CD8 memory response after DIVA^2 in dependence of CD4 help and treatment area. a Mice were immunized twice on both ears with

dithranol (8µg/ear, day 0/7) and IMQ (5% imiquimod [w/w]) together with $OVA_{257-264}$ and or without $OVA_{323-337}$ (day 1/8) or left untreated. The frequencies of SIINFEKL-H-$2K^b$-specific $CD8^+$ T cells were assessed in the blood of the mice on days 7, 14 and 35 (n=7-16). **b** To assess the impact of the immunization area and peptide dose we immunized mice twice with dithranol in petrolatum (8µg/ear, day 0/7) and IMQ (5% imiquimod [w/w]) together with $OVA_{257-264}$ and $OVA_{323-337}$ (30-100 µg/each, day 1/8) or left untreated. $DIVA^2$ treatment was performed on either both ears or on one single ear. In a third group, the two immunizations were split between the ears (d0/1, left ear; d7/8 right ear). The frequencies of SIINFEKL-H-$2K^b$-specific $CD8^+$ T cells were assed in the blood of the mice on days 7, 14 and 35 (n=4-8). Depicted are means + SD. Statistical analysis Kruscal Wallis test with Dunn's post-test (*$p < 0.05$; ns = not significant).
